# The retinal and perceived locus of fixation in the human visual system

**DOI:** 10.1167/jov.21.11.9

**Published:** 2021-10-13

**Authors:** Markku Kilpeläinen, Nicole M. Putnam, Kavitha Ratnam, Austin Roorda

**Affiliations:** 1 Department of Psychology and Logopedics, University of Helsinki, Helsinki, Finland; 2 Arizona College of Optometry, Midwestern University, Glendale, AZ, USA; 3 Facebook Reality Labs, Redmond, WA, USA; 4 Herbert Wertheim School of Optometry and Vision Science, University of California, Berkeley, Berkeley, CA, USA

**Keywords:** fovea, retina, cone photoreceptors, fixation, adaptive optics

## Abstract

Due to the dramatic difference in spatial resolution between the central fovea and the surrounding retinal regions, accurate fixation on important objects is critical for humans. It is known that the preferred retinal location (PRL) for fixation of healthy human observers rarely coincides with the retinal location with the highest cone density. It is not currently known, however, whether the PRL is consistent within an observer or is subject to fluctuations and, moreover, whether observers’ subjective fixation location coincides with the PRL. We studied whether the PRL changes between days. We used an adaptive optics scanning laser ophthalmoscope to project a Maltese cross fixation target on an observer's retina and continuously imaged the exact retinal location of the target. We found that observers consistently use the same PRL across days, regardless of how much the PRL is displaced from the cone density peak location. We then showed observers small stimuli near the visual field location on which they fixated, and the observers judged whether or not the stimuli appeared in fixation. Observers’ precision in this task approached that of fixation itself. Observers based their judgment on both the visual scene coordinates and the retinal location of the stimuli. We conclude that the PRL in a normally functioning visual system is fixed, and observers use it as a reference point in judging stimulus locations.

## Introduction

The fovea of the human retina represents an evolutionary adaptation taken to an extreme. The spatial sampling resolution enabled by the dense packing and narrowing of photoreceptors (and the displacement of post-receptoral neurons) within the human fovea is one of the best among all species (Caves, Brandley, & Johnsen, 2018). In fact, the resolution is as good as allowed by the relatively average optics of the human eye (Liang & Williams, 1997; Marcos & Navarro, 1997). However, it is not feasible for such a foveal specialization to span a significantly larger area of the retina (Provis, Dubis, Maddess, & Carroll, 2013). Thus, the photoreceptor density drops extremely steeply when moving away from the center of the fovea. As a result, performance in many different visual tasks deteriorates dramatically if the stimuli fall even slightly outside the fovea (Mäkelä, Näsänen, Rovamo, & Melmoth, 2001; Rossi & Roorda, 2010; Rovamo, Virsu, Laurinen, & Hyvärinen, 1982; Strasburger, Rentschler, & Harvey, 1994; Thibos, 1998).

Due to such a reliance on a small region of the retina, an oculomotor system enabling fast and precise fixation allocation is critical. Indeed, it has been established that human observers make saccades and hold fixation with high precision and consistency (Kowler & Blaser, 1995; Rattle, 1969). The present study addresses two previously unstudied aspects of fixation: the long-term stability of fixation and the perceived direction of fixation.

Considering the importance of the fovea, as well as the tradeoffs involved, it was quite surprising that Putnam, Hofer, Doble, Chen, Carroll, and Williams (2005) first discovered that the preferred retinal location (PRL) of fixation rarely coincides with the cone density peak, a finding that has been independently confirmed in more recent reports (Li, Tiruveedhula, & Roorda, 2010; Reiniger, Domdei, Holz, & Harmening, 2021; Wang, Bensaid, Tiruveedhula, Ma, Ravikumar, & Roorda, 2019; Wilk, Dubis, Cooper, Summerfelt, Dubra, & Carroll, 2017). Whether the location of the PRL and its displacement from the cone density peak are stable or are subject to fluctuations is not currently known. Evidence of PRL plasticity has been provided, especially by studies with patients suffering from retinal diseases that lead to central vision loss. A particularly interesting population in this respect are patients with an operable macular hole. Such patients often first learn to fixate relatively consistently with a retinal location just outside the hole. After surgery, the PRL moves back toward the center of the fovea (Nakabayashi, Fujikado, Ohji, Saito, & Tano, 2000; Tarita-Nistor, González, Mandelcorn, Lillakas, & Steinbach, 2009). A compelling medical condition is not necessary for the adoption of a substantially non-central PRL, however. Healthy observers with an artificial central scotoma can also learn to use a non-central PRL to carry out various visual tasks with surprisingly high performance (Kwon, Nandy, & Tjan, 2013; Varsori, Perez-Fornos, Safran, & Whatham, 2004; Walsh & Liu, 2014). Such findings, suggesting substantial plasticity of the PRL, lead one to think that perhaps the normal foveal PRL is also subject to some fluctuation, even if unaffected by real or artificial central scotomas. In this study, we tested this hypothesis by determining the PRLs in healthy observers on two or three different days, with variable days in between.

The perceived visual direction of visual field objects can be substantially biased by manipulations of the visual scene itself or the observer's eye movements (Bahcall & Kowler, 1999; Matin, 1972; van Ee, Banks, & Backus, 1999). For example, a flash presented shortly after a saccade must be presented in a clearly different retinal location than a presaccadic flash in order for the two flashes to be perceived in the same location. Such findings demonstrate that observers do not base their direction estimates on retinal coordinates alone. It is not currently known whether the actively fixated visual direction is special in that it would be perceived more veridically. We studied here how the subjective fixation location (SFL), the retinal location that produces the sensation of a stimulus being presented “in fixation,” is determined. Is it determined by the retinal location of the stimulus or by the arrangement of stimuli in the visual field, or both? To determine the observers’ SFLs, we showed them stimuli in various locations across their central retinae and asked the observers to judge whether the stimuli appeared in their line of sight (i.e., in fixation).

The present study shows that, regardless of the amount of displacement of the PRL, it remains a fixed property of each observer's visual system, not subject to fluctuations. Further, observers use the PRL, along with visual scene coordinates, to determine the perceived fixation direction.

## Methods

The datasets generated during this study are available at Open Science Framework (osf.io/2rd9z). Additional data and code are available upon request.

### Observers

Seven observers (three female and four male, ages 24–50 years) with normal visual acuity and normal color vision participated in Experiment 1 and five in Experiment 2. Observers 10002L, 10003R, 20094R, and 30002R were authors of the paper, whereas observers 20092L, 20109R, and 20210R were naïve to the purposes of the experiment. All observers had experience as observers in retinal imaging experiments. Pupil dilation and paralysis of accommodation were achieved with one drop each of tropicamide (1%) and phenylephrine (2.5%), administered 15 minutes before the onset of imaging. Viewing was monocular in both experiments. The observers could freely choose the eye they used in viewing. The R or L at the end of the observer number indicates the eye (right or left) that each observer used. All observers used their dominant eye, except for 20092L, for whom the left eye had a much lower prescription value, which is beneficial in imaging. The study adhered to the tenets of the Declaration of Helsinki and was approved by the University of California, Berkeley, Institutional Review Board. Each observer signed a written informed consent.

### Retinal imaging and stimulation

The adaptive optics scanning laser ophthalmoscope (AOSLO) was used to image the retina and to project fixation targets and other visual stimuli to the retina. The principles of the AOSLO have been presented in detail elsewhere (Mozaffari, LaRocca, Jaedicke, Tiruveedhula, & Roorda, 2020; Poonja, Patel, Henry, & Roorda, 2005), but we describe the most relevant features of the current system here. A light beam from a supercontinuum laser (SuperK EXTREME; NKT Photonics, Birkerød, Denmark) focused on the retina is transformed to a raster by a horizontal scanner (scan rate, 16 kHz) and a vertical scanner (30 Hz). Light reflecting back from the eye is automatically descanned by the same two scanners and then directed to a Shack–Hartmann wavefront sensor, which measures the aberrations of the observer's eye, as well as to a photomultiplier tube (Hamamatsu Photonics, Hamamatsu, Japan), which records the intensity of light from each imaged pixel, enabling the reconstruction of a retinal image. In the current study, 680-nm light was used for all imaging and stimulation and 940-nm light for wavefront sensing. The aberrations measured by the wavefront sensor are compensated for by a deformable mirror (7.2-mm diameter, 97-actuator membrane; ALPAO, Montbonnot-Saint-Martin, France).

Decrement stimuli (dark patterns on a red background) can be presented within the imaging raster by rapidly controlling the power of the scanning beam by means of an acousto-optic modulator (Brimrose Corporation of America, Sparks Glencoe, MD). In the current study, all stimuli were (nominally) 100% decrement patterns. Because such stimuli involve a structured omission of light within the raster, the stimulus shows in the video, and the retinal location of the stimulus can be extracted with absolute certainty, frame by frame, from the videos (Rossi & Roorda, 2010).

### Procedure

Experiment 1 consisted of 30-second fixation runs. Each run started with a Maltese cross fixation target (diameter, 5.5 arcmin) appearing in the center of the 0.93° × 0.93° raster. When the observers felt they were fixating the center of the Maltese cross, the experiment operator started recording the fixation run. During the run, the Maltese cross shifted abruptly (shift distance, 5.5–15.6 arcmin) to a new, random location every 2 to 6 seconds, somewhere within a central 11 × 11-arcmin region of the raster. The observer's task was to carefully fixate on the center of the Maltese cross throughout the 30-second run and to quickly shift fixation to the new location after the cross shifted. The aim of the motion was to more thoroughly engage the subject and to measure the PRL in a situation where periods of fixation are interrupted with saccades. Although still far from natural, this setting resembles normal visual behavior more closely than continuously fixating on a completely stationary and predictable target. A video of the observer's retina (with the target location visible) was recorded throughout the fixation run. See Supplementary Movie S1 for a short version of a retinal video of a fixation run. On each experimental day, the observers carried out eight fixation runs. Observers participated in 2 or 3 experimental days. The experimental days for each observer were on average 2.7 days apart (range, 1–7; *SD*, 1.79, excluding the exceptional 206-day separation).

In Experiment 2, each trial proceeded as follows. The observer was instructed to fixate on the center of the raster (1.16° × 1.16°), according to their subjective estimate. The observer could then initiate a trial by pressing a key. After a random delay of 400 to 700 ms, a small black square (2.3 × 2.3-arcmin, distributed over four lines of the raster scan) was presented within a single frame. The location of the square varied pseudorandomly within a central, 22-arcmin-wide, rectangular area. The stimulation (and imaging) sequence of each trial lasted for 1 second. After that, the observer's task was to judge whether the square had appeared along the observer's line of sight (i.e., in fixation) and to choose from three possible responses (“yes,” “maybe,” “no”). After giving the answer with a key stroke, the observer could initiate the next trial. There were 53 trials in one block. Altogether, each observer completed between 1484 and 1749 trials over three experimental sessions.

### Video and eye motion processing

The videos recorded from the experiments were processed offline according to the following process (Stevenson, Roorda, & Kumar, 2010). First, a composite reference frame was created by aligning and summing selected frames of each video such that the reference frame spanned all of the imaged retinal locations of the video. Each frame of the video was then divided into 28 strips, each 9 pixels in height. These strips were registered with the composite reference, which yielded an eye motion trace with an 840-Hz sampling rate.

**Figure 1.**
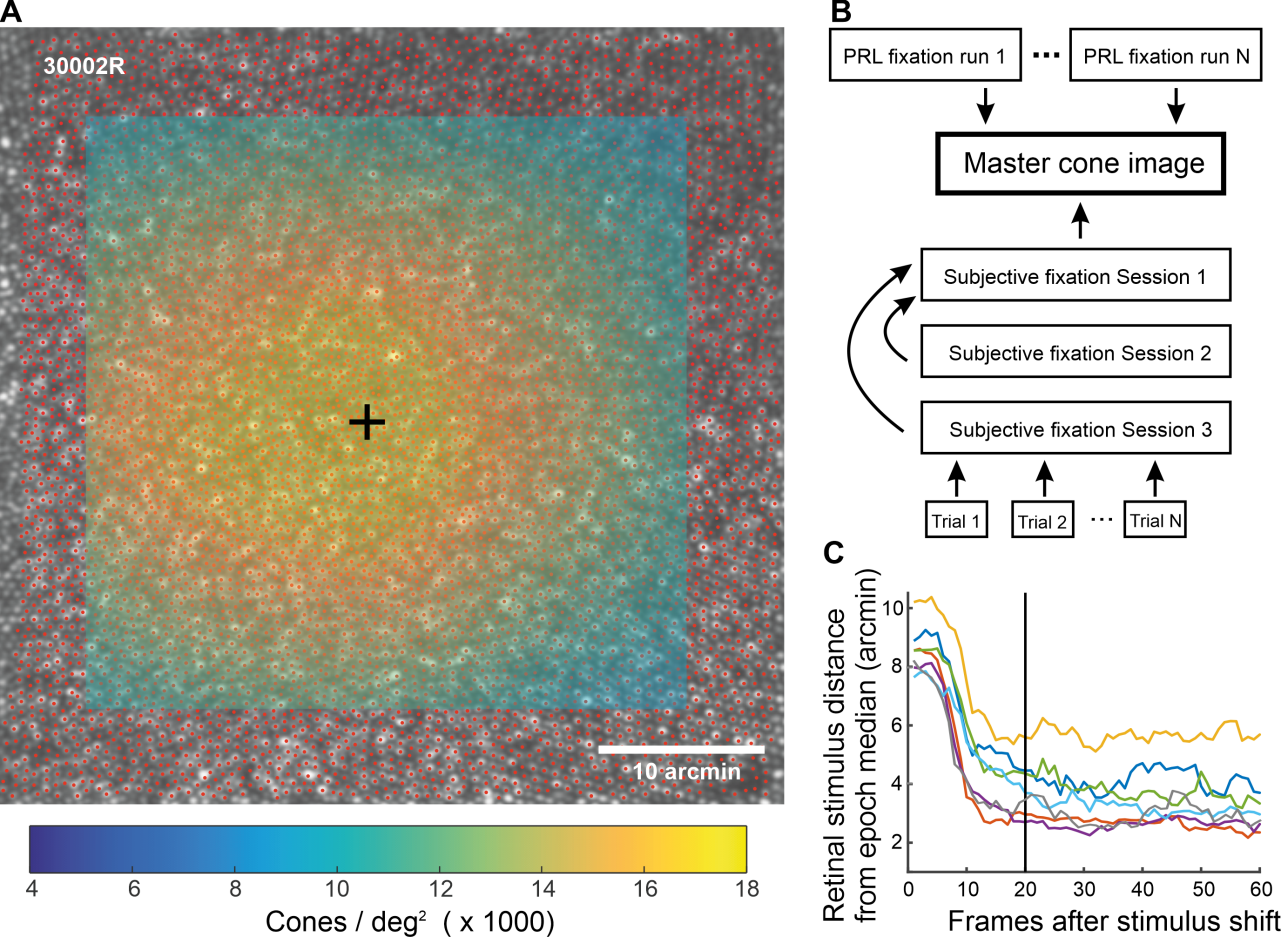
Determining the cone peak, bringing data from all experiments to common coordinates, and removing irrelevant video frames. (A) The cones were marked (red dots) on the master cone image. The map of cone locations was then convolved with a circular window, leading to a map of cone densities (color mapping), from which a peak location (black cross) could be determined. (B) The procedure of bringing all data of the study to the same coordinates. Each box represents a reference frame image. The arrows point to the image to which the other (at arrow origin) was registered to. The MATLAB functions fitgeotrans, imregcorr, and normxcorr2 were used to register the images. (C) Average retinal stimulus location distance from median location as a function of time (in frames) from stimulus shift. Each line represents the data from one observer and is an average over 75 to 90 2-second periods immediately after stimulus shift. The median was calculated over the 2- to 6-second epoch where the stimulus stayed in one location. To exclude frames, where the observer's gaze had not yet moved to the new stimulus location, data from the first 20 frames (∼667 ms) after stimulus shifts was excluded from further analyses (black vertical line). The distance from the median never goes to zero, as the eye hovers around the median location due to fixational eye movements.

To generate a single image to which all data collected in this experiment could be referenced and also to determine the cone density across the fovea, a high-quality master cone image was created in the manner described above but using a 10-second video, while the observer was fixating a black square that was blinking in the center of the raster. The cone locations in the master cone image were manually determined across an area of the central fovea 35 arcmin in diameter. To compute density across the mosaic, the cone locations were converted to a binary map with the same scale as in the cone counting image, where each cone location was assigned a single pixel with a value of 1 (red dots in Figure 1A). This binary image was then convolved with a circular window with a diameter of 8 arcmin. The output of the convolution generates a continuous density map across the image (color mapping in Figure 1A). The point of maximum cone density corresponds to the pixel location of the convolution maximum (black cross in Figure 1A).

**Figure 2.**
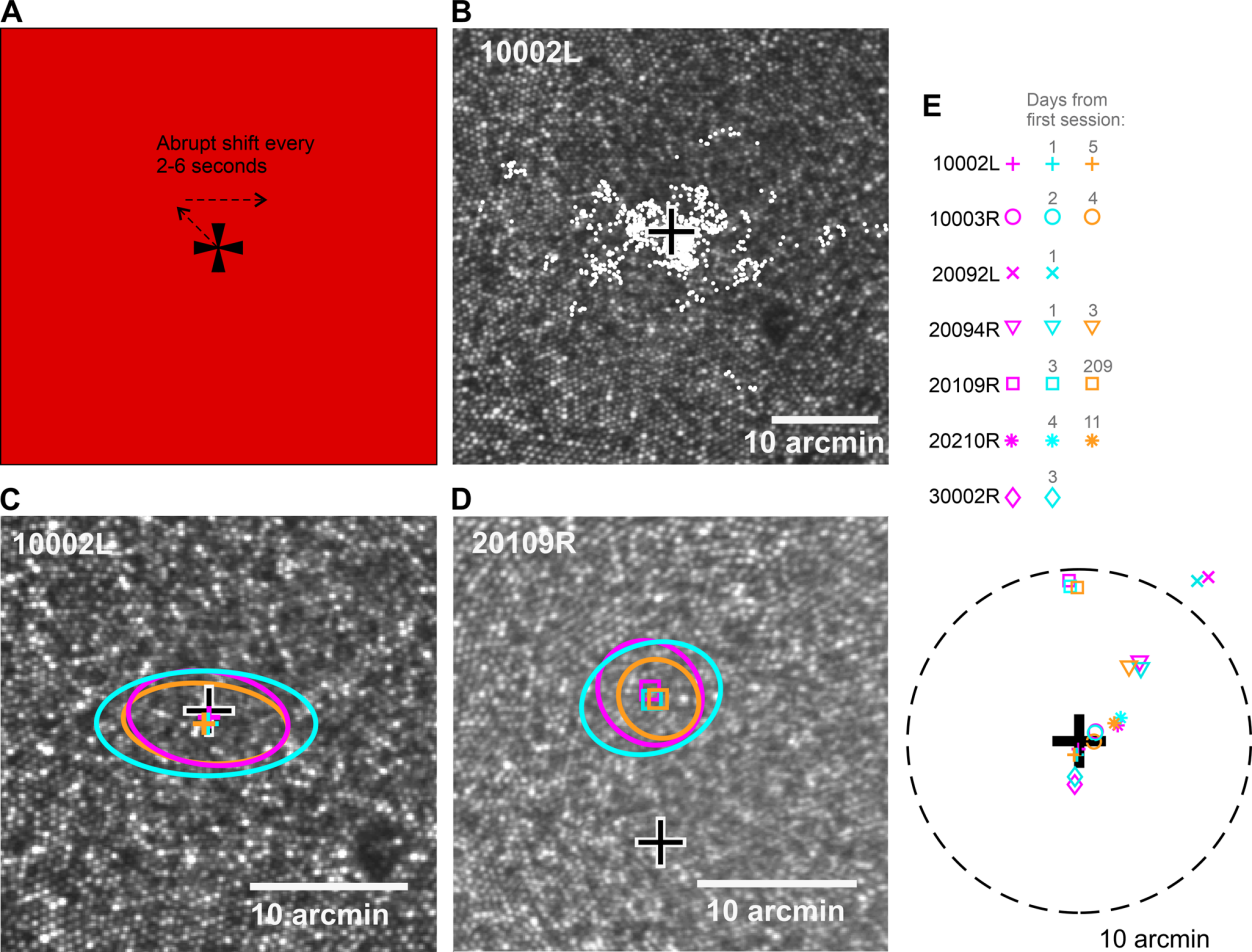
The PRL was stable across days. (A) The stimulus and the task in the PRL experiment (Experiment 1). The observer fixated the Maltese cross, which shifted every 2 to 6 seconds throughout the 30-second fixation run (see also Supplementary Movie S1). (B) Retinal locations of the target from one 30-second video (white markers) plotted on the observer's master cone image. The black-and-white cross indicates the cone density peak. (C, D) Means (markers) and standard deviations (ellipses) of the 2D Gaussians fitted to retinal target locations measured on different days (different colors) for two representative observers. The means (the colored markers) represent the PRLs for different days. (E) The PRLs relative to cone density peak for all observers (see figure legend). Some observers participated on 2 days (two markers), some on 3 days (three markers). The dashed circle signifies a 10-arcmin distance from the cone density peak. Two data points (orange and cyan cross) have been moved 0.08 arcmin apart to make them both visible, as they were on top of each other.

For Experiment 1, the retinal position of the Maltese cross in each video frame was determined by means of cross-correlation at 30 Hz. Note that, as a result, the scatterplot in Figure 2B and Supplementary Movie S1 do not capture motion of the retina during microsaccades which are often completed during a 33-ms frame (Martinez-Conde, Macknik, Troncoso, & Hubel, 2009). We then used the above-mentioned eye motion trace to determine the position of each video frame in the composite reference frame. Finally, to get the stimulus locations from different videos (including from different days) to the same coordinates, the composite reference frames of the different videos were co-registered with the master cone image (see Figure 1B), and the resulting image transformation parameters (translation, scaling, rotation) were applied to the stimulus locations. For each observer and day, the four videos that produced the best co-registration results were included in the data.

Because there is unavoidably a delay between the shift of the Maltese cross and the observer's saccade to the new location, some video frames after each stimulus shift had to be excluded. We plotted the distance between the retinal location of the stimulus in each frame and the median retinal location as a function of time after stimulus shift (Figure 1C). One can observe that after 20 frames (∼667 ms), the saccade has been made; as a result, including all data after that would be likely to introduce very little noise. After removing the 20 first frames and additional removal of frames due to blinks and other image quality degradations, the data included in the analyses had 2191 to 2992 samples (mean, 2710; *SD*, 234.9) per day per observer.

For Experiment 2, the extraction of retinal stimulus locations was done in the same way as for Experiment 1. Bringing data to the coordinates of the master cone image (and thus the same coordinates as the data of Experiment 1) involved some additional steps (see Figure 1B). First, the reference frame of each trial was co-registered with the grand reference frame of the measurement session (produced from a 10-second video taken at the beginning of the session). Second, the grand reference images of sessions 2 and 3 were co-registered with the grand reference image from session 1 to bring all the data of Experiment 2 to the same coordinates. Finally, the grand reference image from session 1 was registered with the master cone image. About 35% of the trials of Experiment 2 could not be included in the data, because either the cross-correlation found a wrong stimulus location or, predominantly, there was a poor alignment between the reference frame of the trial and the grand reference frame of the measurement session. Due to the large number of trials, correcting these by hand was not feasible; however, these errors were expected and were mitigated by collecting a large number of trials.

#### Quantification and statistical analysis

For Experiment 1, the fixation data distributions were fit with two-dimensional (2D) Gaussian distributions. A close look at the measured distributions revealed that they were not strictly Gaussian, but the departure from normality was extreme in only one observer (10003R), with a kurtosis of 10.1 (kurtosis in all other observers was below 7; mean, 2.9; *SD*, 4.39), and there were no cases of skewness over 1 (mean, 0.54; *SD*, 0.28). Thus, because the analysis of the data of Experiment 2 and the comparison between results from the two experiments rely very much on Gaussian distributions, we also fitted the data from Experiment 1 with 2D Gaussian distributions. However, to make sure that the PRLs we are reporting were not specific to a particular fitting method, we also calculated (distribution assumption–free) geometric medians. The location parameters derived in the two ways are in very good agreement, differing on average less than a foveal cone diameter (mean, 0.142 arcmin; *SD*, 0.093; range, 0.03–0.39) (Supplementary Table S1). Differences in the PRLs between two days were analyzed for each observer with the Akaike information criterion (AIC). For observers with three measurements, the two with the largest location difference were used. The AIC values were obtained by fitting 2D Gaussian distributions to the data by means of maximum likelihood estimation with the MATLAB fitgmdist function (MathWorks, Natick, MA). In the simpler model (m1), fixation data from 2 days were fitted with shared parameters only. In the more complex model (m2), the mean (*x*, *y*) coordinates of the two distributions were allowed to differ (shared covariance). The evidence ratio was then calculated from the difference between the AIC values for the more simple model (m1) and the more complex model (m2) as *P*(m1 is best)/*P*(m2 is best), where *P*(m1 is best) = exp(–ΔAIC/2)/[1 + exp(–ΔAIC/2)] and *P*(m2 is best) = 1 – *P*(m1 is best). We additionally analyzed the difference between the same 2 days with a completely distribution assumption–free, two-sample, 2D Kolmogorov–Smirnov (KS) test (Fasano & Franceschini, 1987) with the MATLAB kstest2d function (Lau, 2016). To make sample observations within each day independent, as assumed by the KS test, stimulus locations were averaged across each 2- to 6-second fixation epoch. The range of *D*-statistics of the tests was 0.27 to 0.33 (mean, 0.30; *n* = 25–30 per session per observer) and the range of *p* values was 0.114 to 0.295 (mean, 0.201).

**Figure 3.**
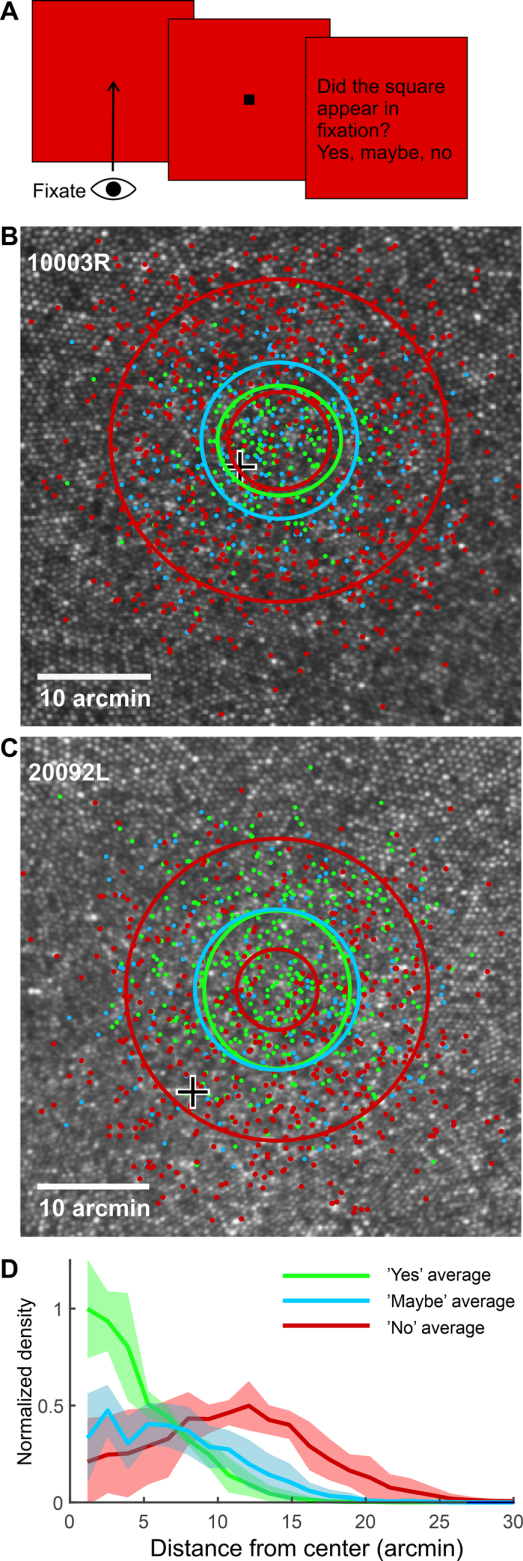
Observers had a strong sense of where they were looking. (A) The stimulus and task in the SFL experiment (Experiment 2). The observer first fixated on the center of the blank raster. Then, a small dark square (not drawn to scale here) appeared near the raster center. The observer then judged whether the stimulus had appeared in the line of sight. (B, C) The retinal locations of all stimuli for two representative observers (dots). The color of each dot indicates the answer given to that stimulus (yes, light green; maybe, light blue; no, red). The ellipses indicate standard deviations of the Gaussian fits to the “yes” and “maybe” data, and a corresponding level (60.6% of maximum) for the difference of Gaussians fit to the “no” data. There are two red ellipses as the density of “no” responses first increases and then decreases when moving outward from the center. The black-and-white cross indicates the cone density peak. (D) Normalized density of different response categories as a function of distance from the center of the fitted function. Shaded areas represent 95% confidence intervals.

For Experiment 2, the SFL for each observer was derived based on all of the responses with the following logic. The probability of “yes” responses should decrease with distance from the SFL following a Gaussian distribution, likewise for the “maybe” responses, although probably with a larger standard deviation. The density of “no” responses should initially increase with distance from the SFL and then decrease, as the stimulated locations become sparser, and were thus modeled with a difference of Gaussians (DoG) function. Because the density of “no” responses cannot be negative, the DoG was scaled to have a minimum of 0. The distributions were fitted to all the responses simultaneously, such that the (*x* and *y*) center location parameters were forced to be the same for the three functions. The standard deviations of the different distributions were allowed to vary independently. The SFL was then given by the common center location parameters of the fitted distributions. The two red ellipses in Figures 3B and 3C correspond to the regions where the DoG function reached 60.6% of maximum (corresponding to 1 *SD* distance from the peak in a single Gaussian function) on the way to the central dip (smaller red ellipse) and to the outer plateau (larger red ellipse).

The SFL lay closer to the PRL than to the cone peak. To test the statistical significance of this distance difference, we conducted a paired-samples *t*-test. Because the distance between the PRL and the cone peak affects how much closer the SFL can be to one of them (if the PRL and the cone peak are at the same location, for example, then the SFL is unavoidably equally far from both), we normalized both the PRL–SFL distance and the cone peak–SFL distance with the PRL–cone peak distance before the *t*-test.

Because observers were instructed to keep fixation close to the center of the raster and the locations of the stimuli were on average very close to the center of the raster, the distance of the stimulus from the raster center and from the PRL are unavoidably correlated (*r* = 0.47; *p* < 0.001). Crucially, though, they also have a significant amount of independent variance. A generalized linear mixed model was used to analyze the contributions of the raster location and the retinal location of the stimulus on the probability of different response alternatives. Because the response variable was an ordinal-scale variable with three possible values, a multinomial logistic link function was used. The analysis was carried out with SPSS Statistics 25 (IBM, Armonk, NY). The repeated-measures nature of the data was incorporated by adding observer as a random factor into the model. In addition, we tested for differences in the proportions of “yes” responses in two circular regions of interest (area coincident with the PRL and an area displaced from the raster center toward a direction opposite the PRL) with a chi-square test for independence, with the following sample sizes: 10003R, *n* = 63 (PRL) and *n* = 43 (opposite); 20094R, *n* = 205 (PRL) and *n* = 225 (opposite); and 20109R, *n* = 160 (PRL) and *n* = 170 (opposite). Sample sizes differed considerably between observers, because (to limit the amount of overlap between regions of interest) the diameter of the analyzed region for each condition was equal to the distance between the raster center and the PRL, in retinal coordinates, which differed among subjects.

## Results

### PRL of fixation was stable across days

In the first experiment, we imaged the retina and the retinal location of a Maltese cross target while observers fixated on the target (see Figure 2A). Our fixation data consist of the retinal location of the target extracted from retinal imaging videos at 30 Hz (Vogel, Arathorn, Roorda, & Parker, 2006). Figure 2B shows the retinal locations of the target extracted from a 30-second video plotted on the cone image.

The PRL of fixation was very stable across the days. Figures 2C and 2D show the means and standard deviations of the 2D Gaussian distributions fitted to the data of two observers. One can see that, whether the PRL was near the cone density peak (large black cross), as for observer 10002L, or farther away, as for observer 20109R, there was very little difference in the mean locations from different days. Figure 2E shows the means from all days and all observers. The largest PRL differences across days for each observer ranged from 0.35 to 0.701 arcmin (mean, 0.538; *SD*, 0.127).

Although it was not one of the main interests of the study, we once again replicated the finding that the PRL was displaced from the cone density peak. The average distance of the PRL from the cone density peak was 4.72 arcmin (*SD*, 4.32), very close to the 5.62-arcmin earlier observed by Li et al. (2010). We also calculated the sampling frequency limits at the cone density peak and the PRL, assuming hexagonal packing (Wang et al., 2019). The average sampling frequency limits were 71.9 cycles per degree (*SD*, 2.48) for the cone density peak and 69.3 cycles per degree (*SD*, 2.74) for the PRL.

To test whether the small differences in PRLs between days might yet be statistically significant, 2D Gaussian distributions were fitted to the data. We used AIC values to compare a simpler model where one distribution was fitted to data from 2 days and another model where the means were estimated separately for the 2 days (see Quantification and Statistical Analysis section). The AIC values produced by the single fit to the data were lower (better) for all observers, as the added parameters produced only small improvements in the fit. Average AIC values were 120,256 (*SD* over observers, 13,834) for the single Gaussian model and 120,262 (*SD*, 13,834) for the two-Gaussian model. The evidence ratio derived from the AIC analysis suggests that the single Gaussian model was on average 22.8 (*SD*, 4.1) times more likely to be the better model than the model with different average locations for the 2 days. Because our data were not strictly Gaussian, we also conducted two-sample KS tests on the same datasets (see Quantification and Statistical Analysis section), which, in line with the AIC analysis, did not indicate a statistically significant difference in the PRL positions for any observers (*p* > 0.1). Although not decisive evidence for either case, both analyses rather support a view of the same PRL across days.

### Observers had a strong sense of where they were looking

In the second experiment, we presented small (2.3 arcmin) and short (one frame) stimuli near the center of the raster while observers maintained fixation near the center of the raster, and we asked the observers to judge, whether the stimulus appeared along their line of sight (i.e., in fixation) or not (see Figure 3A). Observers were able to carry out the task quite reliably. Observers mainly gave the “yes” response only to stimuli landing on a very small region of the retina (green dots in Figures 3B and 3C). Very few “no” responses (red dots), in turn, were given to stimuli landing on the same region.

The SFL for each observer was derived by fitting different functions to the three response categories (see Quantification and statistical analysis section), but with a common center location parameter for all response classes. This center location is our estimate of the SFL. Figures 3B and 3C show all the responses from two representative observers and the fitted functions. The ellipses correspond to 1 *SD* from the mean. Figure 3D shows normalized densities (averaged over five observers) of different responses as a function of distance from the SFL. One can see that the bimodal function was a correct choice for modeling the “no” responses.

Both the location (mean) and dispersion (*SD*) of the SFL could have easily been affected by perceptual mislocalization due to microsaccades occurring near stimulus onset (Hafed, 2013). Considering this possibility, we also fitted the above-described functions to data where all trials with a microsaccade occurring within 250 ms from stimulus onset were filtered out. However, that had virtually no effect on either the location or the distribution of the SFL. Because of that, and the fact that microsaccades were not filtered from Experiment 1 data, we used only unfiltered data in all further analyses of Experiment 2.

### Roles of cone density peak, PRL, and external world stimulus location in observers’ fixation location judgments

**Figure 4.**
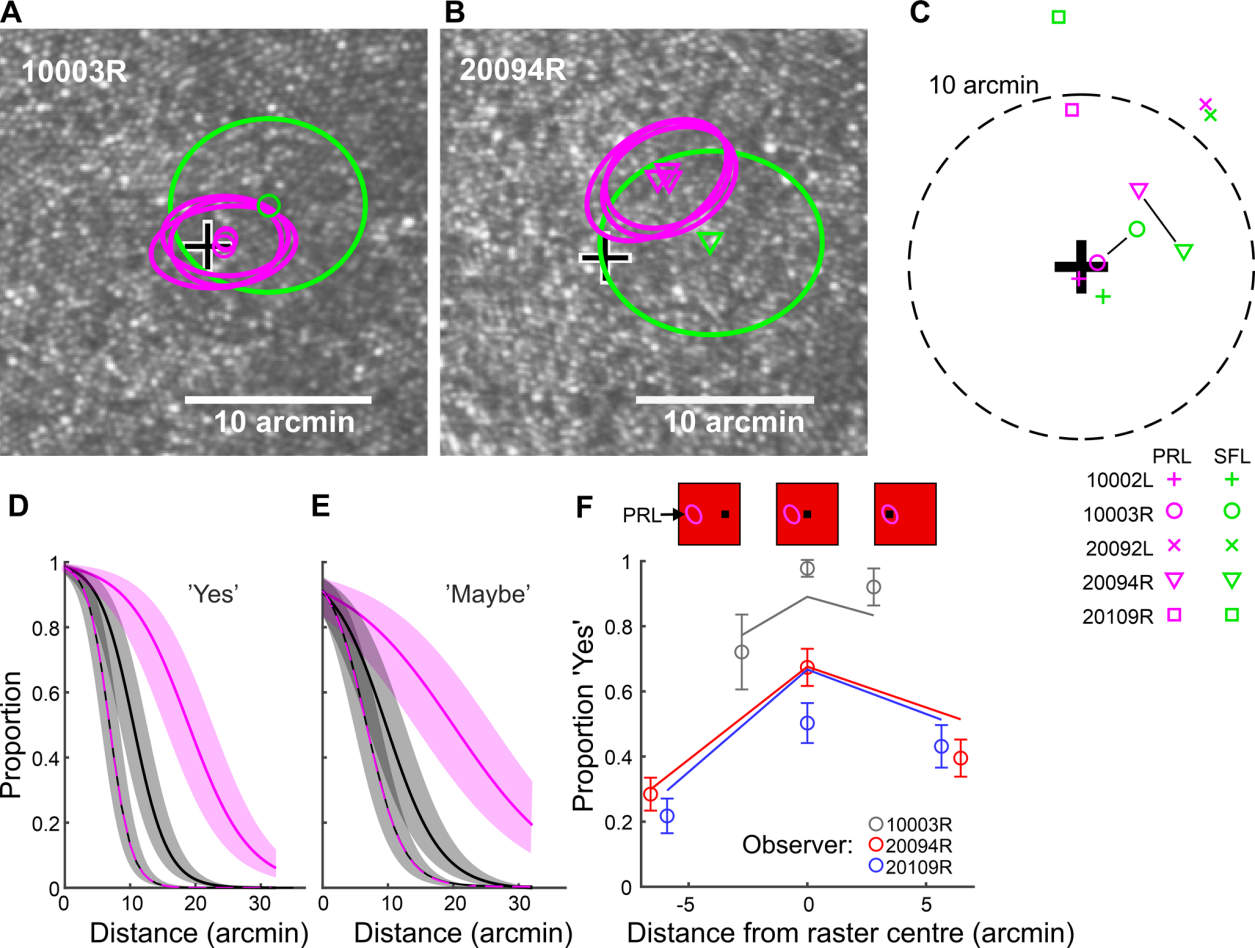
The roles of the cone density peak, the PRL, and the external world stimulus location in the observers’ judgments regarding their fixation locations. (A, B) The cone density peak (large black cross), PRL (magenta markers), and SFL (green markers) of two representative observers. Ellipses indicate standard deviations. (C) All observers’ average PRLs and SFLs relative to each observers’ cone density peak. The SFL has been connected to the observer's PRL with a line in the two cases where it was not the closest one. (D) Proportions of “yes” responses as a function of stimulus distance from the raster center (black line), from the PRL (magenta line), or both simultaneously (black–magenta) as predicted by the generalized linear model. The shaded regions represent the standard error related to the observer effect. (E) As in (D) but for “maybe” responses. (F) Proportion of “yes” responses for different average stimulus locations (see illustrations above markers) for three observers. The “yes” responses were most frequent for all observers when the stimuli were presented near the raster center. The “yes” responses decreased much less, however, if stimuli were displaced from the raster center toward the PRL than to the opposite side of raster center. Error bars represent 95% confidence intervals.

For most observers, the SFL differed somewhat from the PRL, as demonstrated by the distance between the green and magenta symbols in Figures 4A to 4C. However, for all observers, the SFL (green symbols in Figure 4C) was closer to the PRL (magenta symbols; mean distance, 3.07 arcmin; *SD*, 1.93) than to the cone density peak (black cross; mean distance, 7.63 arcmin; *SD*, 5.25) (Figure 4C). This distance difference was statistically significant, *t*(4) = 5.35, *p* = 0.006. Further, both the PRL and SFL were displaced from the cone density peak to a very similar polar angle direction (e.g., both upward for observer 20109R). The correlation between the directions to which the PRL and SFL were displaced from the cone density peak was quite high, *r*(3) = 0.91, *p* = 0.032.

The area where stimuli provoked a “yes” response was larger than the area where observers held the stimuli during the fixation task in Experiment 1. The quadratic mean radius of the *SD* ellipses was on average 5.54 arcmin (*SD*, 0.730) for the “yes” responses and 3.91 arcmin (*SD*, 1.21) for the fixation data. The PRL ellipse was smaller for all observers (see Figures 4A and 4B for examples). The difference was statistically significant, *t*(4) = 5.46, *p* = 0.006. Please note that, although the green ellipses in Figures 4A and 4B indicate the *SD* of “yes” responses only, the location of the SFL (green markers) was determined based on all responses.

It is important to keep in mind that we cannot a priori expect any specific retinal stimulus location to provoke the “in line of sight” sensation, nor can a “yes” response in any specific location be considered objectively correct or incorrect. That said, it is reasonable to hypothesize that stimulus locations near the PRL, on one hand, and near the raster center, on the other hand, might increase the probability of “yes” responses. Note that, although the observer tried to fixate on the center of the raster, the eye constantly moved considerably relative to the raster center (Supplementary Figure S1); hence, stimulus distance from raster center and from the PRL are partially independent. To compare the effects of those two factors on the observers’ judgments, we constructed a generalized mixed linear model with the response (“yes,” “maybe,” “no”) as a dependent variable (see Quantification and Statistical Analysis section). Both main effects, the distance from raster center, *F*(2, 5318) = 62.17, *p* < 0.001, and the distance from the PRL, *F*(2, 5318) = 34.17, *p* < 0.001, were statistically significant. The interaction effect was not significant, *F*(2, 5318 = 0.21, *p* = 0.811. Figures 3D and 3E illustrate the drop in the probability of the stimulus being perceived as “in fixation” with increasing distance of the stimulus from raster center (black curve), with increasing distance of the stimulus from the PRL (magenta curve), and with the distance of the stimulus increasing simultaneously from the PRL and raster center (black-magenta curve).

To illustrate how responses are affected by both the raster position and the retinal location relative to the PRL, we compared the proportions of “yes” responses in three conditions: stimuli presented near the raster center, stimuli displaced from the raster center toward the PRL, and stimuli equally displaced from the raster center but to the opposite direction. Figure 4F shows the model predictions (solid lines) and the data for the three observers (circles; see legend above *x*-axis) for whom the PRL and the average retinal location of the raster center were sufficiently apart that the above-described comparison is meaningful. In line with model predictions, stimuli displaced toward the PRL led to more “yes” responses than stimuli displaced to the opposite direction. The difference was statistically significant for each of the three observers: 10003R, χ^2^(1, *N* = 106) = 7.57 and *p* = 0.006; 20094R, χ^2^(1, *N* = 430) = 5.88 and *p* = 0.015; and 20109R, χ^2^(1, *N* = 330) = 17.25 and *p* < 0.001.

## Discussion

We studied the relationship among the preferred retinal locus (PRL) of fixation and subjective fixation location (SFL) and the location of the cone density peak on the human fovea. The use of AOSLO for stimulation and retinal imaging allowed us to determine very precisely the retinal location of every presented stimulus. We found that, in human observers without any known retinal pathologies, the PRL was very stable across days. Further, the observers were relatively keenly aware of their fixation direction, even when they were not fixating on a particular stimulus.

### PRL across days

Observers consistently used the same retinal location to fixate on a stimulus and the location did not, in general, coincide with the cone density peak. We did not find significant differences in PRLs between days in any of our seven observers. The largest observed difference between two days was 0.7 arcmin, roughly the diameter of two foveal cones. It is a very small difference, considering that a single microsaccade, for example, can move the stimulus across tens of foveal cones (Hauperich, Young, & Smithson, 2020; Nyström, Hansen, Andersson, & Hooge, 2016). Indeed, although Bowers, Gautier, Lin, and Roorda (2021) reported differences in fixational eye movements for different tasks, they did not find differences in the PRL for those tasks. It is also important to note that, in a case where the PRL is, in reality, in the same exact point on two days, any measurement error can only displace the data further apart. No error, in contrast, can move them closer to each other, if they are in the same location to begin with. We conclude that we found no evidence of the PRL moving between days, but long term follow-up studies will be important in the future to test the robustness of this finding.

Because the PRL is constant across days, it is very unlikely that displacement of the PRL from the cone density peak was a result of random, day-to-day fluctuations in the observer's fixation. Rather, each observer's PRL, no matter how far displaced from the cone density peak, was a relatively stable property of the observer's visual system. Why, then, was the PRL in most observers displaced from the retinal location with the highest cone density? One might suspect that the adaptive optics correction to the eye's natural aberrations might have played a role. However, any lateral displacement of the image arising from unlikely prismatic effects of the adaptive optics system must be ruled out because the oculomotor system automatically and immediately compensates for these by refixating on the displaced image. Moreover, at this spatial scale, any meaningful improvement in higher order aberrations along the direction of the PRL compared with nearby locations (such as the cone density peak) is very unlikely. This is because the isoplanatic patch of the eye (the area within which aberrations do not significantly change), spans 1° or more (Bedggood, Daaboul, Ashman, Smith, & Metha, 2008; Nowakowski, Sheehan, Neal, & Goncharov, 2012).

In addition to the cone density peak, the anatomical center of the fovea has been defined based, for example, on the avascular zone and the foveal pit (void of postreceptoral neurons), but those do not coincide with the PRL any more than the cone density peak (Wilk et al., 2017). McGregor et al. (2018) recently defined the foveal center based on the orderly arrangement of ganglion cell somas in macaque retina and found it to be displaced by roughly 8 arcmin from the cone density peak. They studied only one retina, however, and did not measure the PRL. So, if there is a retinal determinant of the PRL, it remains elusive so far.

We suggest that such displacement is more likely to be a consequence of changes that take place during the development of the visual system. Both the maturation of the fovea (Hendrickson, Possin, Vajzovic, & Toth, 2012; Vajzovic et al., 2012) and the development of stereopsis (Giaschi, Narasimhan, Solski, Harrison, & Wilcox, 2013; Norcia & Gerhard, 2015) continue many years (even more than 10) into childhood. Because fixation is in everyday life a binocular process, it might not be possible to base the positions of the eyes solely on cone density. Instead, some small compromises or adaptations are likely to be necessary along the way to enable well-functioning binocular vision. Considering such potentially conflicting developmental pressures, one might argue that our PRLs are quite well placed. The cone (or cones) that the visual system assigned the most attention to during development ends up being surprisingly close to the point of maximum cone density. The PRL displacements observed here were small enough to make the observed (ca. 4%) sampling resolution shortfalls inconsequential, as optical blur in the eye optics reduces spatial resolution in the central fovea by up to 15% (Marcos & Navarro, 1997). For an alternative hypothesis, the reader is referred to Reiniger et al. (2021), who found a consistent superior–nasal PRL displacement and suggested that the displaced PRL might confer more optimized sampling over the binocular visual field.

### Subjective locus of fixation

Observers are keenly aware of whether an object appears in fixation. Although all stimuli were presented within a relatively confined area, stimuli had to appear in a still much more restricted area near the raster center and near the PRL in order to be perceived as in fixation. Although our study lacked an experimental condition where external stimulus coordinates had no role, our mixed model analysis suggests that, for stimuli presented in an identical raster location, observers predominantly judged the stimulus as outside fixation if it landed approximately 0.35° from the PRL. This is not in line with the findings of Wu and Cavanagh (2016). In their experiment, most of the time the observers felt that they could “fixate” on a part of an afterimage that actually lay 1.75° outside their fixation and that they in fact could not move their fixation to (as the afterimage would also move the same amount). The very fact that observers in that study were asked to perform something that is, strictly speaking, impossible makes a direct comparison of their results and ours difficult. Their study and the present one can be considered complementary, as their data show how crude the awareness of fixation location can be when there are no cues about retinal image slip. On the other hand, our study lacks that condition but provides information regarding the roles of an external reference frame and retinal image in the (arguably more natural) condition where both information sources are present. It is a limitation of our study that the reference frame was exceptionally well constrained. However, Rattle (1969) measured fixation accuracy in uniform fields up to 4° in diameter and did not observe fixation accuracy reduction of the scale that would suggest our results are specific to our stimulus conditions.

Poletti, Rucci, and Carrasco (2017) recently showed that the classic perception-boosting effect of a peripheral pre-cue also operates within the fovea. According to our results, however, even the 10-arcmin eccentricity of their experiments was sufficient that observers would rarely consider the stimuli as in fixation. Thus, it remains to be seen whether there can be attentional effects among stimuli that are all perceived to be within the fixation locus.

### Relationship among cone density peak, PRL, and SFL

The SFL was closer to the PRL than to the cone density peak, and both seemed to be displaced to the same direction from the cone density peak. This finding suggests that the displacement of the PRL from the cone density peak is not due to the inability of the oculomotor system to correct a displacement that the visual system can detect.

In three of five observers, there was a clear difference between the PRL and the SFL. Steinman (1965) did observe some differences in fixation position with different-sized fixation targets, but they were not quite as large as those observed here. Regardless of whether the fixation target size (Maltese cross vs. whole imaging raster) was to some extent the cause of the location differences observed here also, these three observers enabled a demonstration of how the judgment of the stimulus location relative to fixation can be affected by the retinal location of the stimulus (in addition to its raster location). The probability of the observers perceiving the stimulus as in fixation was higher when the stimulus was displaced from the raster center toward the PRL rather than the opposite direction (Figure 4F).

More generally, for all five observers in the SFL experiment, the mixed model based on our data suggests that, although the external world reference frames have a strong effect on how the relationship between fixation and stimuli is perceived, the retinal location of the stimulus is also an important cue (Figures 4D and 4E) and would likely be dominant if the external reference frame cue were very weak (e.g., very large homogeneous background). We point out, however, that both cues are usually present in normal visually guided behavior.

Our three-alternative-choice response format was designed to encourage observers to adopt a relatively strict criterion for the high-confidence “yes” response. An objective measure for the observer's criterion and sensitivity in detecting a stimulus in fixation cannot be derived, however, as there is no inherently wrong answer in this task. What we can say is that the standard deviation for “yes” responses was somewhat larger than the standard deviation for fixation for all observers. That is not very surprising, as the oculomotor system tends to refoveate stimuli lying only a few arcminutes from fixation, without the need for a conscious displacement percept (Poletti, Listorti, & Rucci, 2013; Ratnam, 2017). Yet, rather than emphasizing the relatively modest difference, we would argue that the precision of the observers’ SFL estimates came surprisingly close to the precision of fixation itself, considering that the former, unlike the latter, is a skill that is hardly ever needed in everyday life.

## Supplementary Material

Supplement 1

Supplement 2

Supplement 3

## Supplementary material

Supplementary Movie S1. A shortened example video of the fixation task (related to Figure 2). The video shows how the fixation target location is rendered on the video of the observer's retina. We advise the reader to pay attention to the dark spots on the retina to see how the retina follows the movements of the target. The video first plays in normal speed and then in half speed.
