# Supplementary material for: The retinal and perceived locus of fixation in the human visual system
Source: J Vis. 2021 Oct 13;21(11):9. doi: 10.1167/jov.21.11.9 (PMC8525830; doi:10.1167/jov.21.11.9)
Supplement: Supplement 1 [file jovi-21-11-9_s001.pdf]

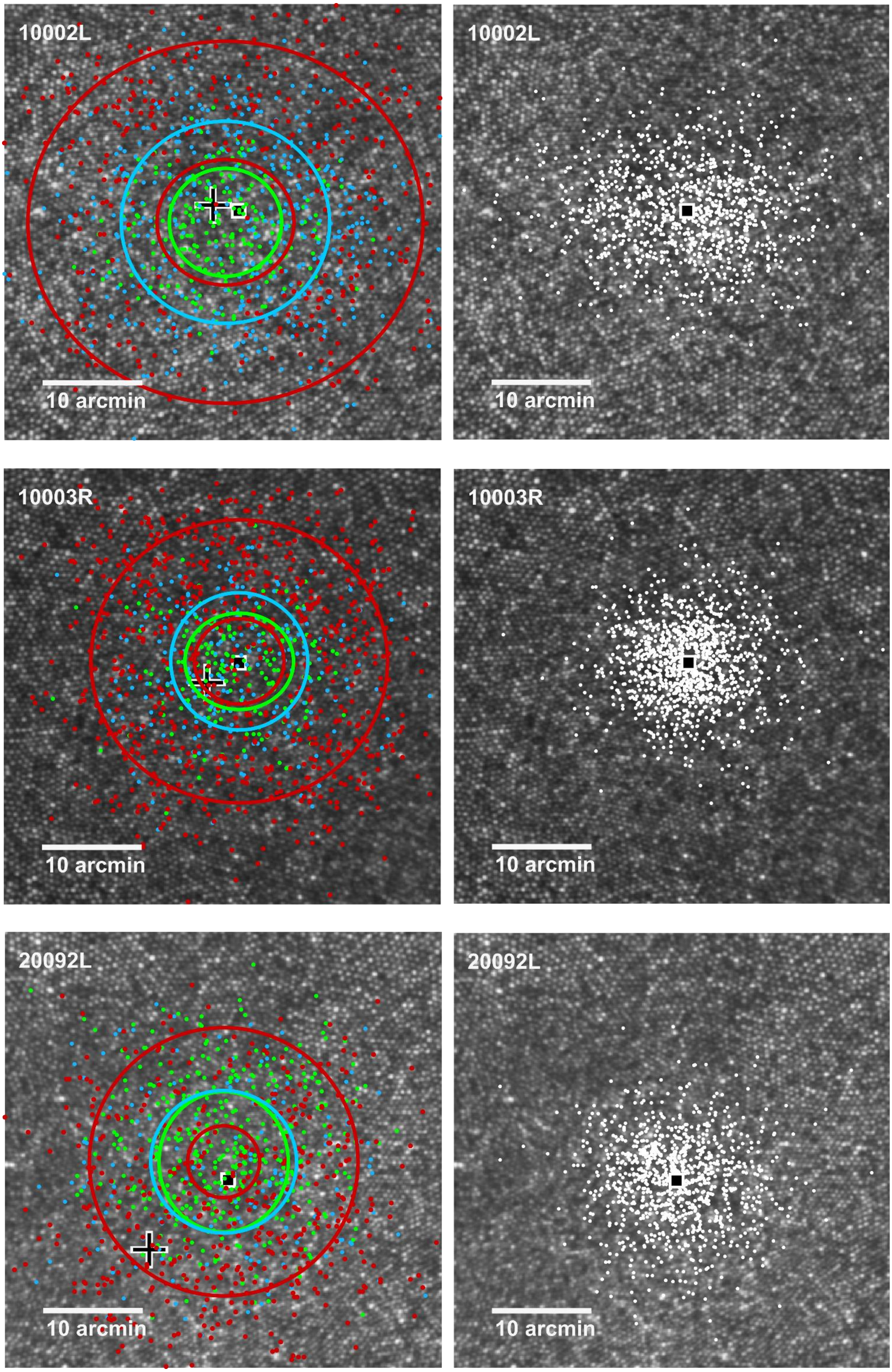
10002L
10 arcmin
10002L
10 arcmin
10003R
10 arcmin
10003R
10 arcmin
20092L
10 arcmin
20092L
10 arcmin

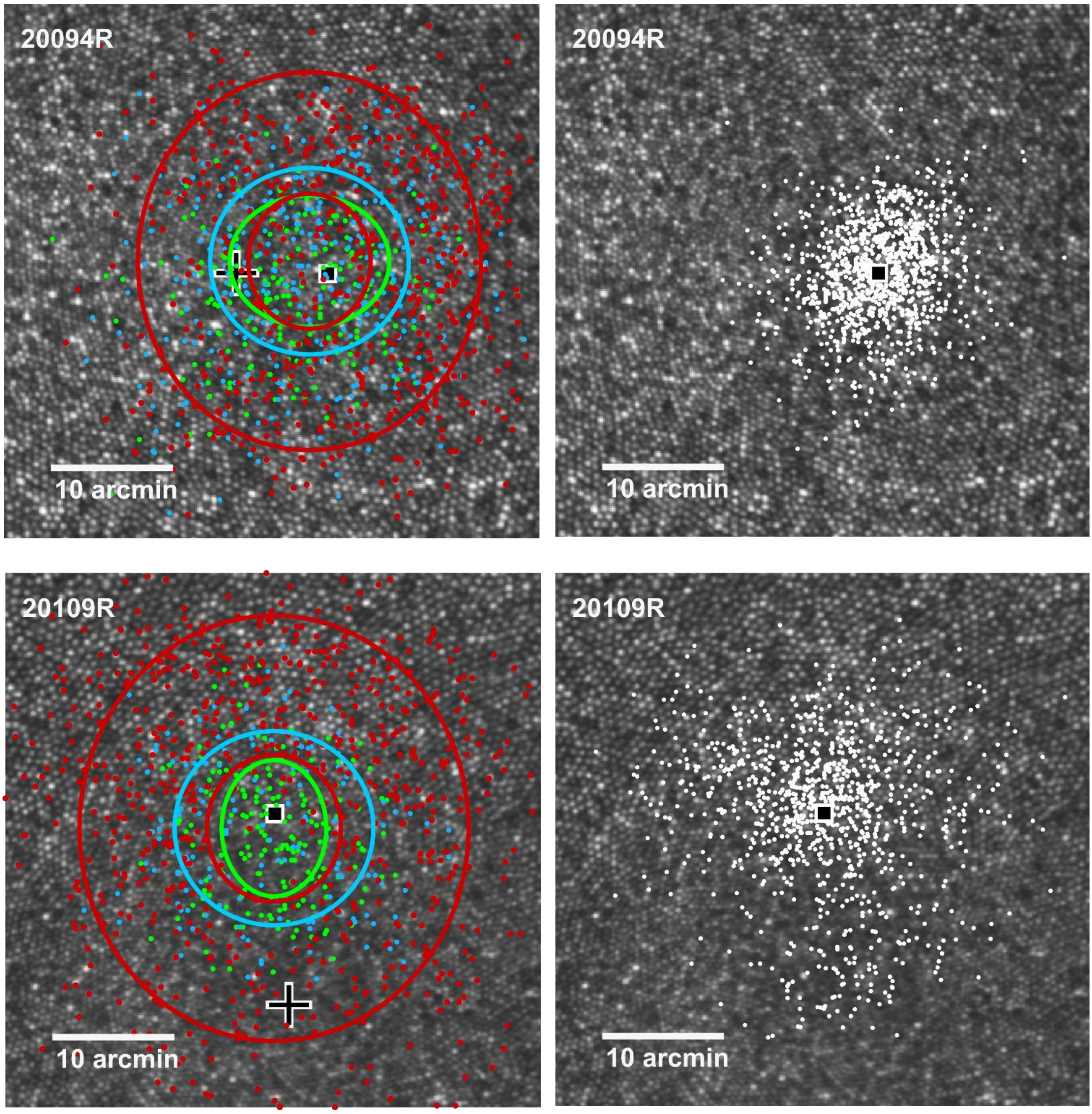

Figure S1. Retinal location of imaging raster center. Related to Figures 3,4. Left, average location of the imaging raster center (black and white square) relative to the cone density peak (black and white cross) and the target stimulus locations and psychophysical responses (color coding as in Figure 3). Right, raster center locations in each trial (white dots), as well as the average location (black and white square).
