# Supplementary material for: The retinal and perceived locus of fixation in the human visual system
Source: J Vis. 2021 Oct 13;21(11):9. doi: 10.1167/jov.21.11.9 (PMC8525830; doi:10.1167/jov.21.11.9)
Supplement: Supplement 2 [file jovi-21-11-9_s002.pdf]

Table S1. The number of days between experimental sessions, and the PRL locations as determined by the fitting of a 2D Gaussian distribution or by calculating the Geometric median. Related to Figure 2E and section QUANTIFICATION AND STATISTICAL ANALYSIS.All values in arcmin, relative to cone peak location.

Observer	Days from first session	Gaussian fit x	Gaussian fit y	Geometric median x	Geometric median y
10002L		-0.048	0.468	0.105	0.607
	1	-0.181	0.797	0.207	0.812
	5	-0.217	0.799	-0.120	0.781
10003R		0.954	-0.339	1.103	-0.411
	2	0.976	-0.420	1.056	-0.482
	4	0.849	0.029	0.767	-0.020
20092L		7.529	-9.544	7.441	-9.454
	1	6.879	-9.319	7.100	-9.190
20094R		3.514	-4.651	3.507	-4.580
	1	3.590	-4.263	3.591	-4.341
	3	2.895	-4.359	3.039	-4.371
20109R		-0.660	-9.358	-0.622	-9.350
	3	-0.594	-9.023	-0.626	-9.232
	209	-0.178	-8.960	-0.468	-8.907
20210R		2.181	-0.932	2.169	-1.012
	4	2.353	-1.371	2.269	-1.297
	11	1.981	-1.060	2.035	-1.057
30002R		-0.270	2.527	-0.286	2.551
	3	-0.259	2.082	-0.195	1.957
